# Different classical hydrogen-bonding patterns in three salicylaldoxime derivatives, 2-HO-4-*X*C_6_H_3_C=NOH (*X* = Me, OH and MeO)

**DOI:** 10.1107/S2056989018013361

**Published:** 2018-09-25

**Authors:** Ligia R. Gomes, Marcus V. N. de Souza, Cristiane F. Da Costa, James L. Wardell, John Nicolson Low

**Affiliations:** a REQUIMTE, Departamento de Química e Bioquímica, Faculdade de Ciências da Universidade do Porto, Rua do Campo Alegre, 687, P-4169-007, Porto, Portugal; b FP-ENAS-Faculdade de Ciências de Saúde, Escola Superior de Saúde da UFP, Universidade Fernando Pessoa, Rua Carlos da Maia, 296, P-4200-150 Porto, Portugal; c Instituto de Tecnologia em Fármacos e Farmanguinhos, Fundação Oswaldo Cruz, 21041-250 Rio de Janeiro, RJ, Brazil; d Department of Chemistry, University of Aberdeen, Meston Walk, Old Aberdeen, AB24 3UE, Scotland

**Keywords:** crystal structure, hydrogen bonding, salicylaldoxime, Hirshfeld surface analysis

## Abstract

The crystal structures of three salicyaldoxime compounds, namely 2-hy­droxy-4-methyl­benzaldehyde oxime, C_8_H_9_NO_2_, **1**, 2,4-di­hydroxy­benzaldehyde oxime, C_7_H_7_NO_3_, **2**, and 2-hy­droxy-4-meth­oxy­benzaldehyde oxime, C_8_H_9_NO_3_, **3**, are discussed. In each compound, the hydroxyl groups are essentially coplanar with their attached phenyl group. The inter­planar angles between the C=N—O moieties of the oxime unit and their attached phenyl rings are 0.08 (9), 1.08 (15) and 6.65 (15)° in **1**, **2** and **3**, respectively. In all three mol­ecules, the 2-hy­droxy group forms an intra­molecular O—H⋯N(oxime) hydrogen bond. In compound (**1**), inter­molecular O—H(oxime)⋯O(hydrox­yl) hydrogen bonds generate *R* ^2^_2_(14) dimers, related by inversion centres. In compound **2**, inter­molecular O—H(oxime)⋯O(4-hy­droxy) hydrogen bonds generate *C*9 chains along the *b*-axis direction, while O—H(4-hydrox­yl)⋯O(2-hydrox­yl) inter­actions form zigzag *C*6 spiral chains along the c-axis direction, generated by a screw axis at 1, *y*, 1/4: the combination of the two chains provides a bimolecular sheet running parallel to the *b* axis, which lies between 0–1/2 *c* and 1/2–1 *c*. In compound **3**, similar *C*9 chains, along the *b-*axis direction are generated by O—H(oxime)⋯O(4-meth­oxy) hydrogen bonds. Further weaker, C—H⋯π (in **1**), π–π (in **2**) and both C—H⋯π and π–π inter­actions (in **3**) further cement the three-dimensional structures. Hirshfeld surface and fingerprint analyses are discussed.

## Abstract 2

The crystal structures of three salicyaldoxime compounds are discussed together with Hirshfeld surface and fingerprint analyses.

## Chemical context   

Aldoximes, *R*CH=NOH, are found in many biologically active compounds (Abele *et al.*, 2008 ▸; Nikitjuka & Jirgensons 2014 ▸), having a diverse range of uses including as anti-tumor agents (Martínez-Pascual *et al.*, 2017 ▸; Qin *et al.*, 2017 ▸; Canario *et al.*, 2018 ▸; Huang *et al.*, 2018 ▸), acaricidal and insecticidal agents (Dai *et al.*, 2017 ▸), thymidine phospho­rylase inhibitors (Zhao *et al.*, 2018 ▸), anti-microbial agents (Yadav *et al.*, 2017 ▸), bacteriocides (Kozlowska *et al.*, 2017 ▸), anti-inflammatory agents (Mohassab *et al.*, 2017 ▸) and in the treatment of nerve-gas poisoning (Lorke *et al.*, 2008 ▸; Voicu *et al.*, 2010 ▸; Katalinić *et al.*, 2017 ▸; Radić *et al.*, 2013 ▸). In the plant kingdom, oximes play a vital role in metabolism (Sørensen *et al.*, 2018 ▸). A specific inter­est in 2-hydroxbenzaldehyde derivatives has arisen regarding their use as ligands for metal complexation (Wood *et al.*, 2006 ▸, 2008*b* ▸).
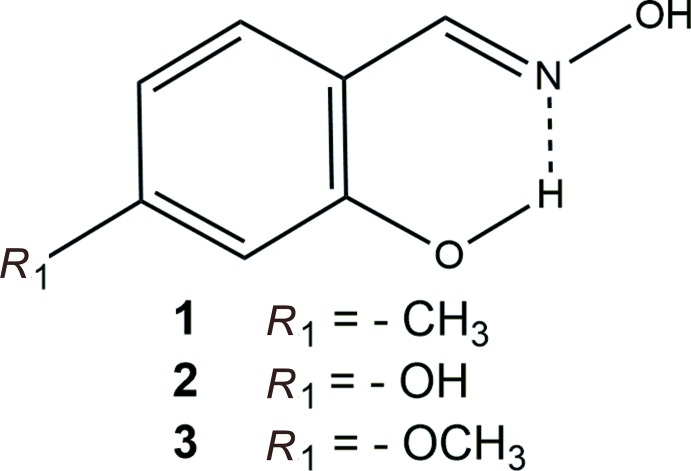


The compounds described herein are all salicylaldoxime derivatives (2-HO-4-*X*-C_6_H_3_-CH=NOH) with different substituents in the 4-position, namely a methyl group, a hy­droxy group and a meth­oxy group, respectively, in compounds, **1**, **2** and **3**. A frequent finding for salicylaldoxime derivatives is the formation of inversion-related 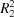
(14) dimers, as concluded from a Cambridge Structural Database survey (CSD Version 5.39, May 2018 update; Groom *et al.*, 2016 ▸). While the structures of many salicylaldoxime derivatives have been reported, the structures of very few compounds with an additional substituent in the 4 position are known.

Compounds **1** and **3** have been shown to have significant activity against *Mycobacterium tuberculosis* ATTC 27294. The full report will be published elsewhere (da Costa *et al.*, 2018 ▸).

## Structural commentary   

**Figure 1:**
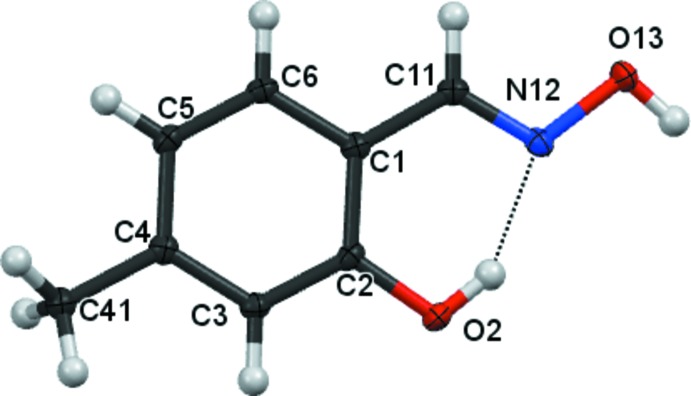
The mol­ecular structure of compound **1**, showing 80% displacement ellipsoids.

**Figure 2:**
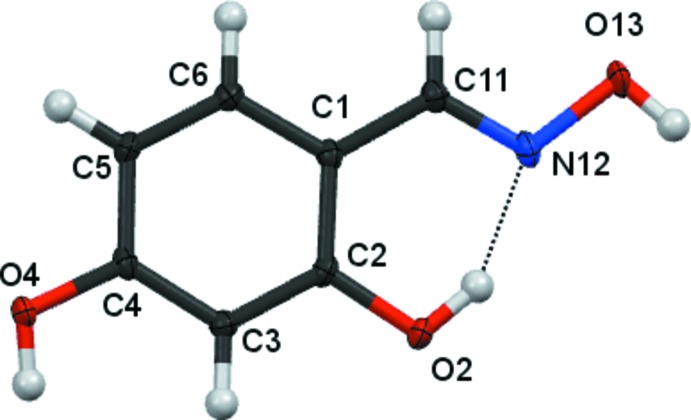
The mol­ecular structure of compound **2**, showing 80% displacement ellipsoids.

There are no unusual features in the mol­ecular structures. Compound **1** (Fig. 1 ▸) crystallizes in the monoclinic space group *P*2_1_/*n* with one mol­ecule in the asymmetric unit. Compounds **2** and **3** crystallize in the monoclinic space group *P*2_1_/*c* with one mol­ecule in the asymmetric unit (Figs. 2 ▸ and 3 ▸), all having an oxime unit with an (*E*) geometry. Bond angles and bond lengths in the phenyl and oxime fragments are all in the expected ranges.

In compound **1**, the hydroxyl group is essentially coplanar with its attached phenyl group [displaced by 0.020 (1) Å], while the inter­planar angle between the C=NO moiety of the oxime unit and the attached phenyl rings is 0.08 (9)°. In compound **2**, the hydroxyl groups lie essentially within the phenyl ring plane [O atoms deviate by −0.003 (1) and 0.006 (1) Å], while the inter­planar angle between the C=NO moiety of the oxime unit and the attached phenyl rings is 1.08 (15)°. In compound **3**, the inter­planar angle between the C=NO moiety of the oxime unit and the attached phenyl rings is 6.65 (15)°.

In all three mol­ecules, an intra­molecular O2—H2⋯N12 hydrogen bond (Tables 1 ▸–3 ▸ ▸) forms a pseudo six-membered ring.

## Supra­molecular features   

### Hydrogen Bonding   

**Figure 4:**
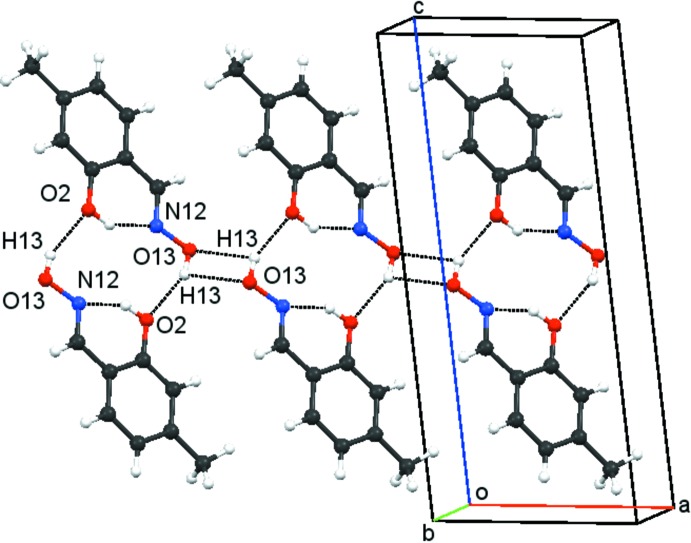
Part of a two-mol­ecule-wide chain in **1** (symmetry codes as in Table 1 ▸).

In the crystal of **1**, mol­ecules are linked by O13—H13 ⋯O2 hydrogen bonds into inversion-related 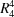
(14) dimers (Table 1 ▸). As stated above, such dimers are the most frequently found arrangement for salicyldoxime derivatives. These 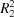
(14), or 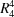
(10) (*via* the intra­molecular hydrogen bond) dimers are linked into two-mol­ecule-wide chains, propagating in the *a*-axis direction by pairs of O13—H13⋯O13 hydrogen bonds, thereby creating 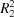
(4) rings, as shown in Fig. 4 ▸. The H13⋯O13 lengths in the O13—H13⋯O13^ii^ hydrogen bond are rather long [2.611 (16) Å] with a small angle of 100.8 (12)°. However, such data fits well with published findings for H_2_O_2_ rings: a recent CSD (Groom *et al.*, 2016 ▸) search revealed more than 500 entries for non-solvated structures having centrosymmetric H_2_O_2_ rings with H—O—H angles of 120° or less and H⋯O distances up to the sum of the van der Waals contact radii, 2.72 Å, of oxygen and hydrogen atoms. The two-mol­ecule-wide chains are further linked into a three-dimensional arrangement by C3—H3⋯*Cg* ^iii^ and C11—H11⋯ *Cg* ^iv^ inter­actions (Table 1 ▸). No π–π inter­actions can be identified.

**Figure 5:**
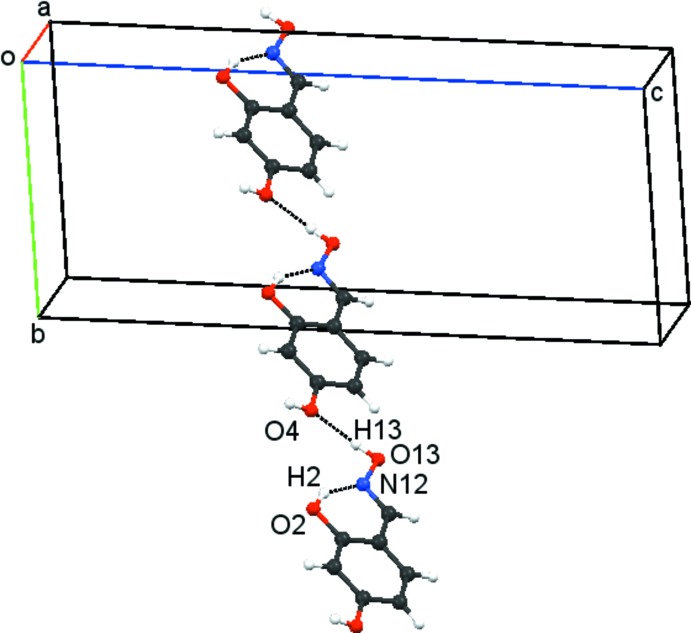
Compound **2**. Part of a *C*9 chain, propagating in the *b-*axis direction, formed by O13—H13⋯O4 hydrogen bonds.

**Figure 6:**
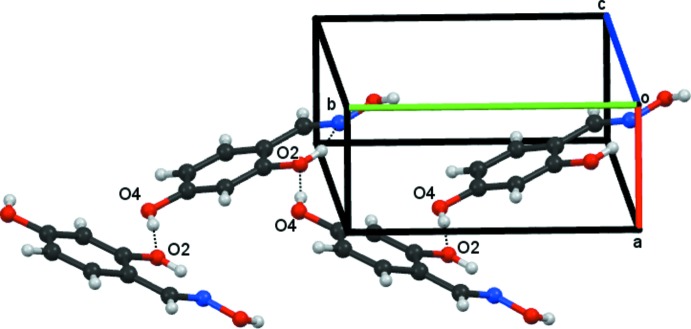
Compound **2**, part of a spiral *C*6 chain formed from O4—H4⋯O2 hydrogen bonds

Compound **2** with two hydroxyl groups, as well as the oxime moiety, produces a much more complex classical hydrogen-bonding arrangement than the one found for compound **1**. The bonding arrangement in **2** can be readily considered to be composed of two elements: a *C*9 chain, generated from O13—H13(oxime)⋯O4(4-hy­droxy)^ii^ hydrogen bonds, propagating in the direction of the *b* axis, see Fig. 5 ▸, and secondly a zigzag *C*6 spiral chain formed from O4—H4⋯O2^i^ hydrogen bonds, see Fig. 6 ▸. The C6 and C9 chains combine to form a bimol­ecular sheet running parallel to the *b* axis which lies between 0–½ *c* and ½–1 *c*. These sheets are further linked by moderately strong π–π stacking inter­actions, involving all the phenyl rings in the sheet: the *Cg*⋯*Cg* separation is 3.7242 (13) Å with a phenyl ring slippage of 1.586 Å. The lack of an 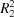
(14) dimer in **2** is apparent and results from the preferential inter­action of the oxime group with the 4-hydroxyl group rather than with the 2-hy­droxy group.

**Figure 7:**
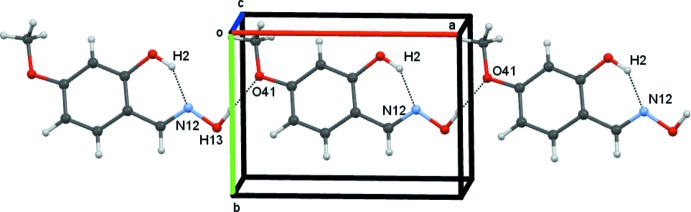
Compound **3**, part of a *C*9 chain of mol­ecules formed by O13—H13⋯O41 hydrogen bonds, propagating along the *a-*axis direction.

In compound **3**, *C*9 chains are generated from O13—H13⋯O41(meth­oxy)^i^ hydrogen bonds, which propagate in the direction of the *b* axis, see Fig. 7 ▸. This chain is similar to that found in compound **2**, but involving the meth­oxy oxygen atom O41 involved instead of the hy­droxy oxygen O4. Inter­estingly, the parameters of the two hydrogen bonds in the chains of compound **2** and **3** are very similar. The chains in compound **3** are linked into a two-dimensional array by C11—H11⋯*Cg* (Table 3 ▸) and π–π inter­actions. The centroid–centroid separation in the π–π inter­action is 3.7926 (12) Å with a phenyl ring slippage of 1.571 Å – again similar parameters are found in the inter­actions of compounds **2** and **3**. The lack of an 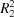
(14) dimer results from the preferential inter­action of the oxime group with the 4-meth­oxy group rather than with the 2-hy­droxy group. The C141—H14*B*⋯O2^ii^ and C3—H3⋯O2^iii^ hydrogen bonds link the molecules into centrosymmetric dimers across the centre of symmetry at (½, 0, ½). The former hydrogen bond forms 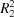
(14) rings, and the latter 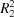
(8) rings. These link anti-parallel *C*9 chains, forming a corrugated ribbon which runs parallel to the *a* axis.

### Hirshfeld Surface Analyses   

**Figure 8:**
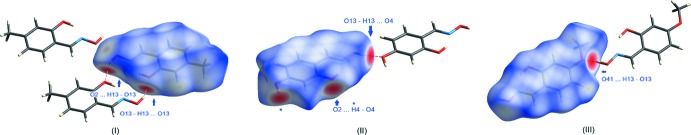
Views of the Hirshfeld surfaces mapped over *d*_norm_ for **1**–**3**. In each case, the red areas relate to classical hydrogen bonds.

**Figure 9:**
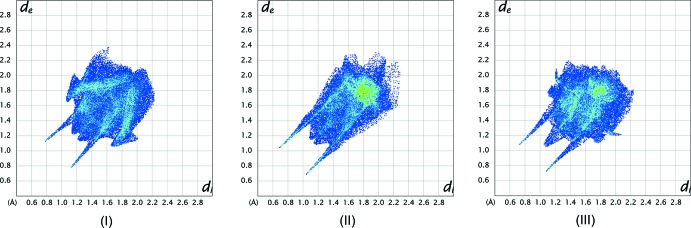
The FP plots for **1**, **2** and **3**. The pair of southwest spikes are due to the O⋯H /H⋯O close contacts. The highest intensity of pixels in the FP plot for **2** at *d*_e_/*d*_i_ = 1.8 Å includes C⋯C contacts.

The Hirshfeld surfaces (Spackman & Jayatilaka, 2009 ▸) and two-dimensional fingerprint (FP) plots (Spackman & McKinnon, 2002 ▸) provide complementary information concerning the inter­molecular inter­actions discussed above. The analyses were generated using *CrystalExplorer3.1* (Wolff *et al.*, 2012 ▸). The Hirshfeld surfaces mapped over *d*_norm_ for **1**–**3** are illustrated in Fig. 8 ▸. The intense red areas on the surfaces correspond to O⋯H close contacts. The less intense red spot on the surface of **1** relates to a O⋯O short contact. The fingerprint plots are shown in Fig. 9 ▸. The percentage contributions to the Hirshfeld surface of the various atom⋯atom contacts shown in Table 4 ▸ are derived from the fingerprint plots.

There are some differences in the percentage of close contacts listed in Table 4 ▸ between the 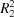
(14) dimer formed by compound **1** and the mol­ecular chains formed by compounds **2** and **3**. Thus compound **1** exhibits the highest percentage of H⋯C/ C⋯H close contacts, but no C⋯C and N⋯O/ O⋯N close contacts, unlike compounds **2** and **3**, and is the only one of the three compounds to have any close O⋯O contacts, albeit a very small percentage. It has to be said that the different substituents, especially the number of hydroxyl units, and other inter­actions, such as C—H⋯π and π–π inter­actions, will have significant effects on the hydrogen-bonding.

## Database survey   

A survey of the Cambridge Structural Database (CSD Version 5.39, May 2018 update; Groom *et al.*, 2016 ▸) of the hydrogen-bonding patterns of oximes confirmed the invariable occurrence for salicylaldoximes, *R*—CH=N—OH (where *R* is a 2-hy­droxy­phenyl derivative) of the formation of intra­molecular O—H⋯NO(oxime) hydrogen bonds involv­ing the *ortho* hydroxyl group. In addition, this hydroxyl group is also most frequently involved in inter­molecular inter­actions producing inversion-related 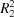
(14) dimers (Smith *et al.*, 2003 ▸; Wood *et al.*, 2006 ▸, 2008*b* ▸). Exceptions include MXSALO [*R* = 2-HO-5-MeOC_6_H_3_, producing a *C*5 chain from O—H(oxime)⋯O(2-hydrox­yl) hydrogen bonds; Pfluger *et al.*, 1978 ▸], YUPSOT [*R* = 2-HO-5-^*t*^Bu-C_6_H_3_, producing a *C*5 chain from O—H(oxime)⋯O(2-hydrox­yl) hydrogen bonds; White *et al.*, 2015*a* ▸], YUPROS [*R* = 2-HO-3-Me-5-(piperin-1-yl-CH_2_)-C_6_H_2_, producing a *C*9 chain from O—H(oxime)⋯N(piperin­yl) hydrogen bonds; White *et al.*, 2015*b* ▸] and XUSPIL [*R* = 2-HO-3-(piperin-1-ylmeth­yl)-5-^*t*^Bu-C_6_H_2_, producing a *C*9 chain from O—H(oxime)⋯N(piperin­yl) hydrogen bonds; Forgan *et al.*, 2010 ▸].

The compounds 2-HO-3-MeOC_6_H_3_CH=N—OH (ABULIT01–07; Forgan *et al.*, 2007 ▸; Wood *et al.*, 2008*a* ▸) and 2-HO-3-EtOC_6_H_3_CH=N—OH (HAHGAA; Cai, 2011 ▸) both form 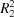
(14) dimers, in contrast to the chain forming 2-HO-4-MeOC_6_H_3_CH=N—OH (this study) and 2-HO-5-MeOC_6_H_3_CH=N—OH (MXSALO; Pfluger *et al.*, 1978 ▸) and 2-HO-5-^*t*^BuOC_6_H_3_CH=N—OH (YUPSOT; White *et al.*, 2015*a* ▸).

An earlier search (Low *et al.*, 2010 ▸) indicated that the most frequently found hydrogen-bonding arrangements for oximes without a 2-hy­droxy­phenyl group are inversion-related 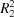
(6) dimers and *C*3 chains.

## Synthesis and crystallization   

The title compounds were prepared from hydroxyl­amine and the corresponding benzaldehyde in methanol in the presence of potassium carbonate and were recrystallized from methanol. Compound **1**, m.p. 378–379 K. Compound **2**, m.p. 451–452 K. Compound **3**, m.p. 410–411 K.

## Refinement details   

Crystal data, data collection and structure refinement details are summarized in Table 5 ▸. All hydroxyl H atoms were refined isotropically. Those attached to C atoms were refined as riding atoms with C—H = 0.95–0.98 Å and *U*_iso_(H) = 1.2–1.5*U*_iso_(C).

## Supplementary Material

Crystal structure: contains datablock(s) 1, 2, 3, global. DOI: 10.1107/S2056989018013361/qm2128sup1.cif

Structure factors: contains datablock(s) 1. DOI: 10.1107/S2056989018013361/qm21281sup2.hkl

Structure factors: contains datablock(s) 2. DOI: 10.1107/S2056989018013361/qm21282sup3.hkl

Structure factors: contains datablock(s) 3. DOI: 10.1107/S2056989018013361/qm21283sup4.hkl

Supporting information file. DOI: 10.1107/S2056989018013361/qm21281sup5.cml

Supporting information file. DOI: 10.1107/S2056989018013361/qm21282sup6.cml

Supporting information file. DOI: 10.1107/S2056989018013361/qm21283sup7.cml

CCDC references: 1868656, 1868655, 1868654

Additional supporting information:  crystallographic information; 3D view; checkCIF report

## Figures and Tables

**Figure 3:**
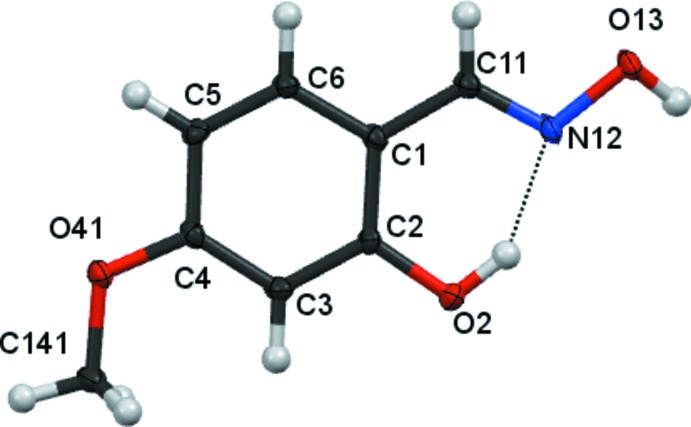
The mol­ecular structure of compound **3**, showing 80% displacement ellipsoids.

**Table 1:** Hydrogen-bond geometry (Å, °) for **1** [Chem scheme1] *Cg* is the centroid of the C1–C6 ring.

*D*—H⋯*A*	*D*—H	H⋯*A*	*D*⋯*A*	*D*—H⋯*A*
O2—H2⋯N12	0.879 (18)	1.814 (18)	2.6066 (10)	149.0 (15)
O13—H13⋯O2^i^	0.857 (17)	2.019 (17)	2.8132 (9)	153.7 (15)
O13—H13⋯O13^ii^	0.857 (17)	2.611 (16)	2.8961 (14)	100.8 (12)
C3—H3⋯*Cg* ^iii^	0.95	2.71	3.4577 (9)	136
C11—H11⋯*Cg* ^iv^	0.95	2.73	3.4910 (9)	138

Symmetry codes: (i) 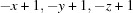; (ii) 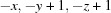; (iii) 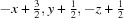; (iv) 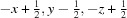.

**Table 2:** Hydrogen-bond geometry (Å, °) for **2** [Chem scheme1]

*D*—H⋯*A*	*D*—H	H⋯*A*	*D*⋯*A*	*D*—H⋯*A*
O2—H2⋯N12	0.91 (3)	1.77 (3)	2.5899 (17)	150 (2)
O4—H4⋯O2^i^	0.86 (2)	1.85 (2)	2.7062 (16)	174 (2)
O13—H13⋯O4^ii^	0.86 (3)	1.90 (3)	2.7583 (16)	171 (2)

Symmetry codes: (i) 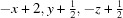; (ii) 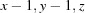.

**Table 3:** Hydrogen-bond geometry (Å, °) for **3** [Chem scheme1] *Cg* is the centroid of the C1–C6 ring.

*D*—H⋯*A*	*D*—H	H⋯*A*	*D*⋯*A*	*D*—H⋯*A*
O2—H2⋯N12	0.92 (3)	1.81 (3)	2.6518 (19)	152 (2)
O13—H13⋯O41^i^	0.91 (3)	1.89 (3)	2.7829 (18)	169 (3)
C141—H14*B*⋯O2^ii^	0.98	2.62	3.412 (2)	138
C3—H3⋯O2^ii^	0.95	2.70	3.570 (2)	154
C11—H11⋯*Cg* ^iii^	0.95	2.89	3.4524 (6)	128

Symmetry codes: (i) 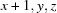; (ii) 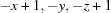; (iii) 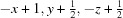.

**Table 4:** Percentages of atom–atom contacts for compounds **1**–**3**

Compound	**1**	**2**	**3**
H⋯H	42.7	36.9	41.5
H⋯O/O⋯H	21.4	33.8	27.9
H⋯C/C⋯H	29.1	10.0	15.5
H⋯N/N⋯H	5.4	2.9	4.1
C⋯C	–	10.8	5.8
O⋯C/C⋯O	1.2	2.2	3.1
N⋯O/O⋯N	–	2.0	0.7
N⋯C/C⋯N	–	–	–
O⋯O	0.2	–	–

**Table 5:** Experimental details

	**1**	**2**	**3**
Crystal data			
Chemical formula	C_8_H_9_NO_2_	C_7_H_7_NO_3_	C_8_H_9_NO_3_
*M*_r_	151.16	153.14	167.16
Crystal system, space group	Monoclinic, *P*2_1_/*n*	Monoclinic, *P*2_1_/*c*	Monoclinic, *P*2_1_/*c*
Temperature (K)	100	100	100
*a*, *b*, *c* (Å)	6.5507 (2), 7.2523 (2), 15.5478 (4)	3.7241 (1), 8.6902 (2), 20.7570 (5)	9.3591 (13), 6.2634 (7), 13.6260 (2)
β (°)	96.737 (3)	92.501 (2)	108.636 (16)
*V* (Å^3^)	733.54 (4)	671.12 (3)	756.87 (15)
*Z*	4	4	4
Radiation type	Mo *K*α	Mo *K*α	Mo *K*α
μ (mm^−1^)	0.10	0.12	0.11
Crystal size (mm)	0.25 × 0.15 × 0.02	0.20 × 0.10 × 0.05	0.15 × 0.05 × 0.01

Data collection			
Diffractometer	Rigaku FRE+ AFC12 with HyPix 6000 detector	Rigaku FRE+ AFC12 with HyPix 6000 detector	Rigaku FRE+ AFC12 with HyPix 6000 detector
Absorption correction	Multi-scan (*CrysAlis PRO* ; Rigaku OD, 2017 ▸)	Multi-scan (*CrysAlis PRO* ; Rigaku OD, 2017 ▸)	Multi-scan (*CrysAlis PRO*; Rigaku OD, 2017 ▸)
*T*_min_, *T*_max_	0.742, 1.000	0.654, 1.000	0.305, 1.000
No. of measured, independent and observed [*I* > 2σ(*I*)] reflections	16323, 1696, 1560	29482, 1537, 1482	5525, 1686, 1323
*R*_int_	0.024	0.039	0.060
(sin θ/λ)_max_ (Å^−1^)	0.649	0.649	0.648

Refinement			
*R*[*F* ^2^ > 2σ(*F* ^2^)], *wR*(*F* ^2^), *S*	0.032, 0.100, 1.08	0.040, 0.092, 0.86	0.049, 0.158, 1.01
No. of reflections	1696	1537	1686
No. of parameters	109	113	118
H-atom treatment	H atoms treated by a mixture of independent and constrained refinement	H atoms treated by a mixture of independent and constrained refinement	H atoms treated by a mixture of independent and constrained refinement
Δρ_max_, Δρ_min_ (e Å^−3^)	0.33, −0.20	0.38, −0.21	0.26, −0.29

Computer programs: *CrysAlis PRO* (Rigaku OD, 2017 ▸), *OSCAIL* (McArdle *et al.*, 2004 ▸), *SHELXT* (Sheldrick, 2015*a* ▸), *ShelXle* (Hübschle *et al.*, 2011 ▸), *SHELXL2017/1* (Sheldrick, 2015*b* ▸), *Mercury* (Macrae *et al.*, 2006 ▸) and *PLATON* (Spek, 2009 ▸).

## supplementary crystallographic information

### 2-Hydroxy-4-methylbenzaldehyde oxime (1) Crystal data

**Table:**

C_8_H_9_NO_2_	*F*(000) = 320
*M**_r_* = 151.16	*D*_x_ = 1.369 Mg m^−^^3^
Monoclinic, *P*2_1_/*n*	Mo *K*α radiation, λ = 0.71075 Å
*a* = 6.5507 (2) Å	Cell parameters from 8222 reflections
*b* = 7.2523 (2) Å	θ = 3.1–31.9°
*c* = 15.5478 (4) Å	µ = 0.10 mm^−^^1^
β = 96.737 (3)°	*T* = 100 K
*V* = 733.54 (4) Å^3^	Plate, brown
*Z* = 4	0.25 × 0.15 × 0.02 mm

### 2-Hydroxy-4-methylbenzaldehyde oxime (1) Data collection

**Table:**

Rigaku FRE+ AFC12 with HyPix 6000 detector diffractometer	1696 independent reflections
Radiation source: Rotating Anode, Rigaku FRE+	1560 reflections with *I* > 2σ(*I*)
Confocal mirrors, VHF Varimax monochromator	*R*_int_ = 0.024
Detector resolution: 10 pixels mm^-1^	θ_max_ = 27.5°, θ_min_ = 2.6°
profile data from ω–scans	*h* = −8→8
Absorption correction: multi-scan (CrysAlisPro ; Rigaku OD, 2017)	*k* = −9→9
*T*_min_ = 0.742, *T*_max_ = 1.000	*l* = −20→20
16323 measured reflections	

### 2-Hydroxy-4-methylbenzaldehyde oxime (1) Refinement

**Table:**

Refinement on *F*^2^	0 restraints
Least-squares matrix: full	Hydrogen site location: mixed
*R*[*F*^2^ > 2σ(*F*^2^)] = 0.032	H atoms treated by a mixture of independent and constrained refinement
*wR*(*F*^2^) = 0.100	*w* = 1/[σ^2^(*F*_o_^2^) + (0.0569*P*)^2^ + 0.1857*P*] where *P* = (*F*_o_^2^ + 2*F*_c_^2^)/3
*S* = 1.08	(Δ/σ)_max_ < 0.001
1696 reflections	Δρ_max_ = 0.33 e Å^−^^3^
109 parameters	Δρ_min_ = −0.20 e Å^−^^3^

### 2-Hydroxy-4-methylbenzaldehyde oxime (1) Special details

**Table:**

Geometry. All esds (except the esd in the dihedral angle between two l.s. planes) are estimated using the full covariance matrix. The cell esds are taken into account individually in the estimation of esds in distances, angles and torsion angles; correlations between esds in cell parameters are only used when they are defined by crystal symmetry. An approximate (isotropic) treatment of cell esds is used for estimating esds involving l.s. planes.

### 2-Hydroxy-4-methylbenzaldehyde oxime (1) Fractional atomic coordinates and isotropic or equivalent isotropic displacement parameters (Å^2^)

**Table:**

	*x*	*y*	*z*	*U*_iso_*/*U*_eq_	
O2	0.62147 (10)	0.55213 (9)	0.38936 (4)	0.01715 (19)	
H2	0.520 (3)	0.516 (2)	0.4176 (11)	0.046 (4)*	
O13	0.11027 (10)	0.35295 (10)	0.46255 (4)	0.01893 (19)	
H13	0.155 (2)	0.389 (2)	0.5139 (11)	0.041 (4)*	
N12	0.27030 (11)	0.40720 (11)	0.41612 (5)	0.01501 (19)	
C1	0.38496 (13)	0.41353 (12)	0.27677 (5)	0.0129 (2)	
C2	0.57244 (13)	0.50209 (12)	0.30486 (5)	0.0131 (2)	
C3	0.71163 (13)	0.54354 (12)	0.24671 (6)	0.0139 (2)	
H3	0.837332	0.603208	0.266799	0.017*	
C4	0.66904 (13)	0.49864 (12)	0.15934 (6)	0.0137 (2)	
C5	0.48296 (14)	0.41041 (12)	0.13081 (6)	0.0144 (2)	
H5	0.451750	0.379046	0.071398	0.017*	
C6	0.34460 (13)	0.36875 (12)	0.18861 (6)	0.0139 (2)	
H6	0.219471	0.308503	0.168196	0.017*	
C11	0.23470 (13)	0.36707 (12)	0.33550 (6)	0.0139 (2)	
H11	0.110265	0.306989	0.313953	0.017*	
C41	0.81701 (14)	0.54974 (13)	0.09625 (6)	0.0173 (2)	
H41A	0.814596	0.454650	0.051360	0.026*	
H41B	0.956069	0.559362	0.126945	0.026*	
H41C	0.776779	0.668498	0.069346	0.026*	

### 2-Hydroxy-4-methylbenzaldehyde oxime (1) Atomic displacement parameters (Å^2^)

**Table:**

	*U*^11^	*U*^22^	*U*^33^	*U*^12^	*U*^13^	*U*^23^
O2	0.0175 (3)	0.0221 (4)	0.0115 (3)	−0.0050 (3)	0.0003 (2)	−0.0029 (2)
O13	0.0156 (3)	0.0260 (4)	0.0162 (3)	−0.0046 (3)	0.0059 (3)	−0.0030 (3)
N12	0.0137 (4)	0.0158 (4)	0.0162 (4)	−0.0005 (3)	0.0048 (3)	0.0001 (3)
C1	0.0133 (4)	0.0110 (4)	0.0141 (4)	0.0013 (3)	0.0009 (3)	0.0004 (3)
C2	0.0159 (4)	0.0114 (4)	0.0115 (4)	0.0016 (3)	−0.0009 (3)	−0.0006 (3)
C3	0.0135 (4)	0.0124 (4)	0.0153 (4)	−0.0004 (3)	−0.0001 (3)	0.0000 (3)
C4	0.0152 (4)	0.0112 (4)	0.0147 (4)	0.0020 (3)	0.0016 (3)	0.0009 (3)
C5	0.0166 (4)	0.0137 (4)	0.0124 (4)	0.0015 (3)	−0.0009 (3)	−0.0008 (3)
C6	0.0133 (4)	0.0122 (4)	0.0153 (4)	0.0005 (3)	−0.0016 (3)	−0.0009 (3)
C11	0.0132 (4)	0.0118 (4)	0.0164 (4)	0.0007 (3)	0.0007 (3)	−0.0005 (3)
C41	0.0177 (4)	0.0192 (4)	0.0153 (4)	−0.0016 (3)	0.0029 (3)	0.0000 (3)

### 2-Hydroxy-4-methylbenzaldehyde oxime (1) Geometric parameters (Å, º)

**Table:**

O2—C2	1.3645 (10)	C3—H3	0.9500
O2—H2	0.879 (18)	C4—C5	1.4018 (13)
O13—N12	1.3973 (9)	C4—C41	1.5041 (12)
O13—H13	0.857 (17)	C5—C6	1.3826 (12)
N12—C11	1.2812 (11)	C5—H5	0.9500
C1—C6	1.4033 (12)	C6—H6	0.9500
C1—C2	1.4091 (12)	C11—H11	0.9500
C1—C11	1.4584 (12)	C41—H41A	0.9800
C2—C3	1.3902 (12)	C41—H41B	0.9800
C3—C4	1.3928 (12)	C41—H41C	0.9800
			
C2—O2—H2	107.2 (11)	C6—C5—C4	120.40 (8)
N12—O13—H13	101.6 (11)	C6—C5—H5	119.8
C11—N12—O13	112.33 (7)	C4—C5—H5	119.8
C6—C1—C2	117.75 (8)	C5—C6—C1	121.44 (8)
C6—C1—C11	119.63 (8)	C5—C6—H6	119.3
C2—C1—C11	122.61 (8)	C1—C6—H6	119.3
O2—C2—C3	118.06 (8)	N12—C11—C1	120.08 (8)
O2—C2—C1	121.18 (8)	N12—C11—H11	120.0
C3—C2—C1	120.75 (8)	C1—C11—H11	120.0
C2—C3—C4	120.80 (8)	C4—C41—H41A	109.5
C2—C3—H3	119.6	C4—C41—H41B	109.5
C4—C3—H3	119.6	H41A—C41—H41B	109.5
C3—C4—C5	118.86 (8)	C4—C41—H41C	109.5
C3—C4—C41	120.51 (8)	H41A—C41—H41C	109.5
C5—C4—C41	120.60 (8)	H41B—C41—H41C	109.5
			
C6—C1—C2—O2	179.14 (7)	C3—C4—C5—C6	−0.06 (13)
C11—C1—C2—O2	−1.14 (14)	C41—C4—C5—C6	−178.02 (8)
C6—C1—C2—C3	0.13 (13)	C4—C5—C6—C1	0.24 (14)
C11—C1—C2—C3	179.86 (8)	C2—C1—C6—C5	−0.28 (13)
O2—C2—C3—C4	−178.99 (7)	C11—C1—C6—C5	179.99 (7)
C1—C2—C3—C4	0.04 (14)	O13—N12—C11—C1	179.95 (7)
C2—C3—C4—C5	−0.08 (13)	C6—C1—C11—N12	179.91 (8)
C2—C3—C4—C41	177.88 (7)	C2—C1—C11—N12	0.19 (14)

### 2-Hydroxy-4-methylbenzaldehyde oxime (1) Hydrogen-bond geometry (Å, º)

Cg is the centroid of the C1–C6 ring.

**Table:**

*D*—H···*A*	*D*—H	H···*A*	*D*···*A*	*D*—H···*A*
O2—H2···N12	0.879 (18)	1.814 (18)	2.6066 (10)	149.0 (15)
O13—H13···O2^i^	0.857 (17)	2.019 (17)	2.8132 (9)	153.7 (15)
O13—H13···O13^ii^	0.857 (17)	2.611 (16)	2.8961 (14)	100.8 (12)
C3—H3···*Cg*^iii^	0.95	2.71	3.4577 (9)	136
C11—H11···*Cg*^iv^	0.95	2.73	3.4910 (9)	138

Symmetry codes: (i) −*x*+1, −*y*+1, −*z*+1; (ii) −*x*, −*y*+1, −*z*+1; (iii) −*x*+3/2, *y*+1/2, −*z*+1/2; (iv) −*x*+1/2, *y*−1/2, −*z*+1/2.

### 2,4-Dihydroxybenzaldehyde oxime (2) Crystal data

**Table:**

C_7_H_7_NO_3_	*F*(000) = 320
*M**_r_* = 153.14	*D*_x_ = 1.516 Mg m^−^^3^
Monoclinic, *P*2_1_/*c*	Mo *K*α radiation, λ = 0.71075 Å
*a* = 3.7241 (1) Å	Cell parameters from 13388 reflections
*b* = 8.6902 (2) Å	θ = 1.9–32.1°
*c* = 20.7570 (5) Å	µ = 0.12 mm^−^^1^
β = 92.501 (2)°	*T* = 100 K
*V* = 671.12 (3) Å^3^	Block, colourless
*Z* = 4	0.20 × 0.10 × 0.05 mm

### 2,4-Dihydroxybenzaldehyde oxime (2) Data collection

**Table:**

Rigaku FRE+ AFC12 with HyPix 6000 detector diffractometer	1537 independent reflections
Radiation source: Rotating Anode, Rigaku FRE+	1482 reflections with *I* > 2σ(*I*)
Confocal mirrors, VHF Varimax monochromator	*R*_int_ = 0.039
Detector resolution: 10 pixels mm^-1^	θ_max_ = 27.5°, θ_min_ = 2.0°
profile data from ω–scans	*h* = −4→4
Absorption correction: multi-scan (CrysAlisPro ; Rigaku OD, 2017)	*k* = −11→11
*T*_min_ = 0.654, *T*_max_ = 1.000	*l* = −26→26
29482 measured reflections	

### 2,4-Dihydroxybenzaldehyde oxime (2) Refinement

**Table:**

Refinement on *F*^2^	0 restraints
Least-squares matrix: full	Hydrogen site location: mixed
*R*[*F*^2^ > 2σ(*F*^2^)] = 0.040	H atoms treated by a mixture of independent and constrained refinement
*wR*(*F*^2^) = 0.092	*w* = 1/[σ^2^(*F*_o_^2^) + (0.0229*P*)^2^ + 1.3357*P*] where *P* = (*F*_o_^2^ + 2*F*_c_^2^)/3
*S* = 0.86	(Δ/σ)_max_ < 0.001
1537 reflections	Δρ_max_ = 0.38 e Å^−^^3^
113 parameters	Δρ_min_ = −0.21 e Å^−^^3^

### 2,4-Dihydroxybenzaldehyde oxime (2) Special details

**Table:**

Geometry. All esds (except the esd in the dihedral angle between two l.s. planes) are estimated using the full covariance matrix. The cell esds are taken into account individually in the estimation of esds in distances, angles and torsion angles; correlations between esds in cell parameters are only used when they are defined by crystal symmetry. An approximate (isotropic) treatment of cell esds is used for estimating esds involving l.s. planes.
Refinement. Refined as a 2-component twin.

### 2,4-Dihydroxybenzaldehyde oxime (2) Fractional atomic coordinates and isotropic or equivalent isotropic displacement parameters (Å^2^)

**Table:**

	*x*	*y*	*z*	*U*_iso_*/*U*_eq_	
O2	0.6604 (3)	0.13314 (13)	0.28983 (5)	0.0175 (3)	
H2	0.568 (7)	0.056 (3)	0.3130 (12)	0.045 (7)*	
O4	1.0469 (3)	0.64910 (12)	0.32704 (5)	0.0167 (3)	
H4	1.132 (6)	0.639 (3)	0.2893 (11)	0.031 (6)*	
O13	0.2536 (3)	−0.13686 (13)	0.41952 (6)	0.0213 (3)	
H13	0.208 (7)	−0.200 (3)	0.3880 (12)	0.043 (7)*	
N12	0.3984 (4)	−0.01346 (15)	0.38573 (6)	0.0161 (3)	
C1	0.6052 (4)	0.24418 (16)	0.39524 (7)	0.0125 (3)	
C2	0.7061 (4)	0.25485 (16)	0.33098 (7)	0.0129 (3)	
C3	0.8530 (4)	0.38889 (17)	0.30720 (7)	0.0133 (3)	
H3	0.9193	0.3946	0.2636	0.016*	
C4	0.9020 (4)	0.51454 (17)	0.34786 (7)	0.0133 (3)	
C5	0.8047 (4)	0.50773 (17)	0.41191 (7)	0.0148 (3)	
H5	0.8379	0.5943	0.4395	0.018*	
C6	0.6596 (4)	0.37332 (17)	0.43460 (7)	0.0141 (3)	
H6	0.5947	0.3683	0.4783	0.017*	
C11	0.4474 (4)	0.10553 (17)	0.42134 (7)	0.0144 (3)	
H11	0.3805	0.1034	0.4650	0.017*	

### 2,4-Dihydroxybenzaldehyde oxime (2) Atomic displacement parameters (Å^2^)

**Table:**

	*U*^11^	*U*^22^	*U*^33^	*U*^12^	*U*^13^	*U*^23^
O2	0.0273 (6)	0.0106 (5)	0.0150 (5)	−0.0041 (5)	0.0047 (4)	−0.0036 (4)
O4	0.0249 (6)	0.0096 (5)	0.0161 (5)	−0.0052 (4)	0.0051 (4)	0.0002 (4)
O13	0.0335 (7)	0.0110 (5)	0.0194 (6)	−0.0095 (5)	0.0025 (5)	0.0019 (4)
N12	0.0189 (6)	0.0103 (6)	0.0191 (6)	−0.0029 (5)	0.0010 (5)	0.0030 (5)
C1	0.0127 (7)	0.0096 (6)	0.0151 (7)	0.0003 (5)	0.0005 (5)	0.0003 (5)
C2	0.0142 (7)	0.0101 (6)	0.0143 (7)	0.0008 (5)	−0.0003 (5)	−0.0023 (5)
C3	0.0149 (7)	0.0123 (7)	0.0126 (6)	0.0003 (6)	0.0020 (5)	0.0000 (5)
C4	0.0135 (7)	0.0087 (6)	0.0176 (7)	−0.0005 (5)	0.0009 (5)	0.0020 (5)
C5	0.0180 (7)	0.0106 (7)	0.0158 (7)	−0.0010 (6)	0.0015 (6)	−0.0027 (5)
C6	0.0158 (7)	0.0132 (7)	0.0135 (7)	−0.0005 (6)	0.0021 (5)	−0.0010 (5)
C11	0.0158 (7)	0.0117 (7)	0.0156 (7)	−0.0003 (6)	0.0003 (5)	0.0019 (5)

### 2,4-Dihydroxybenzaldehyde oxime (2) Geometric parameters (Å, º)

**Table:**

O2—C2	1.3655 (17)	C1—C11	1.456 (2)
O2—H2	0.91 (3)	C2—C3	1.387 (2)
O4—C4	1.3660 (17)	C3—C4	1.387 (2)
O4—H4	0.86 (2)	C3—H3	0.9500
O13—N12	1.4020 (16)	C4—C5	1.394 (2)
O13—H13	0.86 (3)	C5—C6	1.378 (2)
N12—C11	1.280 (2)	C5—H5	0.9500
C1—C6	1.398 (2)	C6—H6	0.9500
C1—C2	1.405 (2)	C11—H11	0.9500
			
C2—O2—H2	106.5 (16)	O4—C4—C3	121.64 (13)
C4—O4—H4	111.3 (15)	O4—C4—C5	117.43 (13)
N12—O13—H13	99.9 (16)	C3—C4—C5	120.93 (14)
C11—N12—O13	112.18 (13)	C6—C5—C4	118.93 (14)
C6—C1—C2	117.65 (13)	C6—C5—H5	120.5
C6—C1—C11	119.85 (13)	C4—C5—H5	120.5
C2—C1—C11	122.50 (13)	C5—C6—C1	121.98 (14)
O2—C2—C3	117.93 (13)	C5—C6—H6	119.0
O2—C2—C1	120.77 (13)	C1—C6—H6	119.0
C3—C2—C1	121.30 (13)	N12—C11—C1	120.25 (14)
C2—C3—C4	119.22 (13)	N12—C11—H11	119.9
C2—C3—H3	120.4	C1—C11—H11	119.9
C4—C3—H3	120.4		
			
C6—C1—C2—O2	−179.97 (14)	O4—C4—C5—C6	−179.63 (14)
C11—C1—C2—O2	−0.2 (2)	C3—C4—C5—C6	0.3 (2)
C6—C1—C2—C3	−0.3 (2)	C4—C5—C6—C1	−0.4 (2)
C11—C1—C2—C3	179.41 (14)	C2—C1—C6—C5	0.4 (2)
O2—C2—C3—C4	179.88 (13)	C11—C1—C6—C5	−179.33 (14)
C1—C2—C3—C4	0.2 (2)	O13—N12—C11—C1	178.31 (12)
C2—C3—C4—O4	179.71 (14)	C6—C1—C11—N12	−179.63 (14)
C2—C3—C4—C5	−0.2 (2)	C2—C1—C11—N12	0.6 (2)

### 2,4-Dihydroxybenzaldehyde oxime (2) Hydrogen-bond geometry (Å, º)

**Table:**

*D*—H···*A*	*D*—H	H···*A*	*D*···*A*	*D*—H···*A*
O2—H2···N12	0.91 (3)	1.77 (3)	2.5899 (17)	150 (2)
O4—H4···O2^i^	0.86 (2)	1.85 (2)	2.7062 (16)	174 (2)
O13—H13···O4^ii^	0.86 (3)	1.90 (3)	2.7583 (16)	171 (2)

Symmetry codes: (i) −*x*+2, *y*+1/2, −*z*+1/2; (ii) *x*−1, *y*−1, *z*.

### 2-Hydroxy-4-methoxybenzaldehyde oxime (3) Crystal data

**Table:**

C_8_H_9_NO_3_	*F*(000) = 352
*M**_r_* = 167.16	*D*_x_ = 1.467 Mg m^−^^3^
Monoclinic, *P*2_1_/*c*	Mo *K*α radiation, λ = 0.71075 Å
*a* = 9.3591 (13) Å	Cell parameters from 1379 reflections
*b* = 6.2634 (7) Å	θ = 3.3–30.2°
*c* = 13.6260 (2) Å	µ = 0.11 mm^−^^1^
β = 108.636 (16)°	*T* = 100 K
*V* = 756.87 (15) Å^3^	Plate, colourless
*Z* = 4	0.15 × 0.05 × 0.01 mm

### 2-Hydroxy-4-methoxybenzaldehyde oxime (3) Data collection

**Table:**

Rigaku FRE+ AFC12 with HyPix 6000 detector diffractometer	1686 independent reflections
Radiation source: Rotating Anode, Rigaku FRE+	1323 reflections with *I* > 2σ(*I*)
Confocal mirrors, VHF Varimax monochromator	*R*_int_ = 0.060
Detector resolution: 10 pixels mm^-1^	θ_max_ = 27.4°, θ_min_ = 2.3°
profile data from ω–scans	*h* = −11→12
Absorption correction: multi-scan (CrysAlisPro; Rigaku OD, 2017)	*k* = −7→7
*T*_min_ = 0.305, *T*_max_ = 1.000	*l* = −17→17
5525 measured reflections	

### 2-Hydroxy-4-methoxybenzaldehyde oxime (3) Refinement

**Table:**

Refinement on *F*^2^	0 restraints
Least-squares matrix: full	Hydrogen site location: mixed
*R*[*F*^2^ > 2σ(*F*^2^)] = 0.049	H atoms treated by a mixture of independent and constrained refinement
*wR*(*F*^2^) = 0.158	*w* = 1/[σ^2^(*F*_o_^2^) + (0.1063*P*)^2^] where *P* = (*F*_o_^2^ + 2*F*_c_^2^)/3
*S* = 1.00	(Δ/σ)_max_ < 0.001
1686 reflections	Δρ_max_ = 0.26 e Å^−^^3^
118 parameters	Δρ_min_ = −0.29 e Å^−^^3^

### 2-Hydroxy-4-methoxybenzaldehyde oxime (3) Special details

**Table:**

Geometry. All esds (except the esd in the dihedral angle between two l.s. planes) are estimated using the full covariance matrix. The cell esds are taken into account individually in the estimation of esds in distances, angles and torsion angles; correlations between esds in cell parameters are only used when they are defined by crystal symmetry. An approximate (isotropic) treatment of cell esds is used for estimating esds involving l.s. planes.

### 2-Hydroxy-4-methoxybenzaldehyde oxime (3) Fractional atomic coordinates and isotropic or equivalent isotropic displacement parameters (Å^2^)

**Table:**

	*x*	*y*	*z*	*U*_iso_*/*U*_eq_	
O2	0.63191 (14)	0.1847 (2)	0.42822 (9)	0.0193 (3)	
H2	0.711 (3)	0.265 (5)	0.423 (2)	0.049 (7)*	
O13	0.91351 (14)	0.6293 (2)	0.38674 (10)	0.0228 (4)	
H13	0.981 (3)	0.524 (5)	0.388 (2)	0.062 (9)*	
O41	0.10398 (13)	0.30632 (19)	0.36291 (8)	0.0182 (3)	
N12	0.78766 (16)	0.5076 (2)	0.38782 (11)	0.0175 (4)	
C1	0.52304 (18)	0.5260 (3)	0.36005 (11)	0.0145 (4)	
C2	0.51014 (18)	0.3173 (3)	0.39480 (11)	0.0147 (4)	
C3	0.37212 (18)	0.2374 (3)	0.39667 (12)	0.0158 (4)	
H3	0.365066	0.097118	0.421199	0.019*	
C4	0.24440 (19)	0.3667 (3)	0.36197 (12)	0.0150 (4)	
C5	0.25337 (19)	0.5722 (3)	0.32479 (12)	0.0172 (4)	
H5	0.165354	0.657670	0.299366	0.021*	
C6	0.39104 (19)	0.6495 (3)	0.32543 (12)	0.0158 (4)	
H6	0.397195	0.790761	0.301740	0.019*	
C11	0.66646 (18)	0.6195 (3)	0.36181 (12)	0.0157 (4)	
H11	0.670249	0.765245	0.343458	0.019*	
C141	0.0866 (2)	0.0952 (3)	0.39792 (14)	0.0220 (4)	
H14A	−0.018185	0.074061	0.395637	0.033*	
H14B	0.153667	0.076825	0.469161	0.033*	
H14C	0.112340	−0.009659	0.352899	0.033*	

### 2-Hydroxy-4-methoxybenzaldehyde oxime (3) Atomic displacement parameters (Å^2^)

**Table:**

	*U*^11^	*U*^22^	*U*^33^	*U*^12^	*U*^13^	*U*^23^
O2	0.0191 (7)	0.0148 (6)	0.0240 (7)	0.0044 (5)	0.0068 (5)	0.0056 (5)
O13	0.0187 (6)	0.0164 (7)	0.0360 (8)	−0.0006 (5)	0.0127 (5)	0.0018 (5)
O41	0.0181 (6)	0.0152 (7)	0.0220 (6)	0.0002 (5)	0.0075 (5)	0.0021 (4)
N12	0.0179 (7)	0.0163 (8)	0.0200 (7)	−0.0027 (5)	0.0083 (6)	−0.0003 (5)
C1	0.0200 (8)	0.0122 (8)	0.0122 (8)	−0.0001 (6)	0.0062 (6)	−0.0011 (6)
C2	0.0175 (8)	0.0147 (8)	0.0121 (7)	0.0024 (6)	0.0051 (6)	−0.0005 (6)
C3	0.0216 (9)	0.0118 (8)	0.0149 (8)	0.0013 (6)	0.0073 (6)	0.0007 (6)
C4	0.0180 (8)	0.0155 (9)	0.0119 (7)	−0.0001 (6)	0.0054 (6)	−0.0024 (6)
C5	0.0204 (8)	0.0150 (8)	0.0160 (8)	0.0045 (6)	0.0055 (6)	0.0006 (6)
C6	0.0235 (9)	0.0106 (8)	0.0139 (8)	0.0017 (6)	0.0068 (6)	0.0008 (6)
C11	0.0204 (9)	0.0131 (8)	0.0143 (8)	0.0004 (6)	0.0065 (6)	−0.0004 (5)
C141	0.0225 (9)	0.0144 (9)	0.0290 (9)	−0.0018 (7)	0.0082 (7)	0.0031 (7)

### 2-Hydroxy-4-methoxybenzaldehyde oxime (3) Geometric parameters (Å, º)

**Table:**

O2—C2	1.365 (2)	C3—C4	1.395 (2)
O2—H2	0.92 (3)	C3—H3	0.9500
O13—N12	1.4067 (18)	C4—C5	1.396 (2)
O13—H13	0.91 (3)	C5—C6	1.374 (2)
O41—C4	1.371 (2)	C5—H5	0.9500
O41—C141	1.432 (2)	C6—H6	0.9500
N12—C11	1.283 (2)	C11—H11	0.9500
C1—C2	1.409 (2)	C141—H14A	0.9800
C1—C6	1.405 (2)	C141—H14B	0.9800
C1—C11	1.458 (2)	C141—H14C	0.9800
C2—C3	1.393 (2)		
			
C2—O2—H2	104.4 (17)	C6—C5—C4	119.18 (15)
N12—O13—H13	100.6 (19)	C6—C5—H5	120.4
C4—O41—C141	117.95 (13)	C4—C5—H5	120.4
C11—N12—O13	111.75 (14)	C5—C6—C1	122.04 (15)
C2—C1—C6	117.58 (15)	C5—C6—H6	119.0
C2—C1—C11	123.02 (15)	C1—C6—H6	119.0
C6—C1—C11	119.37 (15)	N12—C11—C1	120.87 (16)
O2—C2—C3	117.11 (15)	N12—C11—H11	119.6
O2—C2—C1	121.63 (15)	C1—C11—H11	119.6
C3—C2—C1	121.26 (15)	O41—C141—H14A	109.5
C4—C3—C2	118.99 (15)	O41—C141—H14B	109.5
C4—C3—H3	120.5	H14A—C141—H14B	109.5
C2—C3—H3	120.5	O41—C141—H14C	109.5
O41—C4—C3	123.83 (15)	H14A—C141—H14C	109.5
O41—C4—C5	115.24 (14)	H14B—C141—H14C	109.5
C3—C4—C5	120.92 (16)		
			
C6—C1—C2—O2	178.92 (13)	C2—C3—C4—C5	0.7 (2)
C11—C1—C2—O2	−3.1 (2)	O41—C4—C5—C6	177.11 (13)
C6—C1—C2—C3	−1.2 (2)	C3—C4—C5—C6	−1.9 (2)
C11—C1—C2—C3	176.75 (14)	C4—C5—C6—C1	1.6 (2)
O2—C2—C3—C4	−179.21 (13)	C2—C1—C6—C5	−0.1 (2)
C1—C2—C3—C4	0.9 (2)	C11—C1—C6—C5	−178.10 (14)
C141—O41—C4—C3	−2.5 (2)	O13—N12—C11—C1	−178.61 (13)
C141—O41—C4—C5	178.56 (14)	C2—C1—C11—N12	5.8 (2)
C2—C3—C4—O41	−178.25 (14)	C6—C1—C11—N12	−176.33 (14)

### 2-Hydroxy-4-methoxybenzaldehyde oxime (3) Hydrogen-bond geometry (Å, º)

Cg is the centroid of the C1–C6 ring.

**Table:**

*D*—H···*A*	*D*—H	H···*A*	*D*···*A*	*D*—H···*A*
O2—H2···N12	0.92 (3)	1.81 (3)	2.6518 (19)	152 (2)
O13—H13···O41^i^	0.91 (3)	1.89 (3)	2.7829 (18)	169 (3)
C141—H14*B*···O2^ii^	0.98	2.62	3.412 (2)	138
C3—H3···O2^ii^	0.95	2.70	3.570 (2)	154
C11—H11···*Cg*^iii^	0.95	2.89	3.4524 (6)	128

Symmetry codes: (i) *x*+1, *y*, *z*; (ii) −*x*+1, −*y*, −*z*+1; (iii) −*x*+1, *y*+1/2, −*z*+1/2.

## References

[bb1] Abele, E., Abele, R. & Lukevics, E. (2008). *Chem. Heterocycl. Cmpd*, **44**, 769–792.

[bb2] Cai, L.-F. (2011). *Z. Kristallogr. New Cryst. Struct.* **226**, 315–316.

[bb3] Canario, C., Silvestre, S., Falcao, A. & Alves, G. (2018). *Curr. Med. Chem.* **25**, 660–686. 10.2174/0929867324666171003115400 28971759

[bb4] Coles, S. J. & Gale, P. A. (2012). *Chem. Sci.* **3**, 683–689.

[bb5] Costa, C. F. da, Lourenço, M. C. S., Coimbra, E. S., Carvalho, G. S., Wardell, J. L., Calixto, S. L., Granato, J. T. & de Souza, M. V. N. (2018). Unpublished observations.

[bb6] Dai, H., Chen, J., Li, G., Ge, S. S., Shi, Y. J., Fang, Y. & Ling, Y. (2017). *Bioorg. Med. Chem. Lett.* **27**, 950–953. 10.1016/j.bmcl.2016.12.083 28108247

[bb7] Forgan, R. S., Davidson, J. E., Fabbiani, F. P. A., Galbraith, S. G., Henderson, D. K., Moggach, S. A., Parsons, S., Tasker, P. A. & White, F. J. (2010). *Dalton Trans.* **39**, 1763–1770. 10.1039/b916877j 20449420

[bb8] Forgan, R. S., Wood, P. A., Campbell, J., Henderson, D. K., McAllister, F. E., Parsons, S., Pidcock, E., Swart, R. M. & Tasker, P. A. (2007). *Chem. Commun.* pp. 4940–4942. 10.1039/b712278k 18361376

[bb9] Groom, C. R., Bruno, I. J., Lightfoot, M. P. & Ward, S. C. (2016). *Acta Cryst.* B**72**, 171–179. 10.1107/S2052520616003954 PMC4822653 27048719

[bb10] Huang, G., Zhao, H. R., Meng, Q. Q., Zhang, Q. J., Dong, J. Y., Zhu, B. Q. & Li, S. S. (2018). *Eur. J. Med. Chem.* **143**, 166–181. 10.1016/j.ejmech.2017.11.031 29174813

[bb11] Hübschle, C. B., Sheldrick, G. M. & Dittrich, B. (2011). *J. Appl. Cryst.* **44**, 1281–1284. 10.1107/S0021889811043202 PMC3246833 22477785

[bb12] Katalinić, M., Zandona, A., Ramić, A., Zorbaz, T., Primožič, I. & Kovarik, Z. (2017). *Molecules*, **22**, No. 1234. 10.3390/molecules22071234 PMC6151989 28737687

[bb13] Kozlowska, J., Potaniec, B., Zarowska, B. & Aniol, M. (2017). *Molecules*, **22** No. 1485. 10.3390/molecules22091485 PMC6151618 28878189

[bb14] Lorke, D. E., Kalasz, H., Petroianu, G. A. & Tekes, K. (2008). *Curr. Med. Chem.* **15**, 743–753. 10.2174/092986708783955563 18393843

[bb15] Low, J. N., Santos, L. M. N. B. F., Lima, C. F. R. A. C., Brandão, P. & Gomes, L. R. (2010). *Eur. J. Chem.* **1**, 61–66.

[bb16] Macrae, C. F., Edgington, P. R., McCabe, P., Pidcock, E., Shields, G. P., Taylor, R., Towler, M. & van de Streek, J. (2006). *J. Appl. Cryst.* **39**, 453–457.

[bb17] Martínez-Pascual, R., Meza-Reyes, S., Vega-Baez, J. L., Merino-Montiel, P., Padrón, J. M., Mendoza, Á. & Montiel-Smith, S. (2017). *Steroids*, **122**, 24–33. 10.1016/j.steroids.2017.03.008 28396219

[bb18] McArdle, P., Gilligan, K., Cunningham, D., Dark, R. & Mahon, M. (2004). *CrystEngComm*, **6**, 303–309.

[bb19] Mohassab, M., Hassan, H. A., Abdelhamid, D., Abdel-Aziz, M., Dalby, K. N. & Kaoud, T. S. (2017). *Bioorg. Chem.* **75**, 242–259. 10.1016/j.bioorg.2017.09.018 29032325

[bb20] Nikitjuka, A. & Jirgensons, A. (2014). *Chem. Heterocycl. C.* **49**, 1544–1559.

[bb21] Pfluger, C. E., Pfluger, M. T. & Brackett, E. B. (1978). *Acta Cryst.* B**34**, 1017–1019.

[bb22] Qin, H. L., Leng, J., Youssif, B. G. M., Amjad, M. W., Raja, M. A. G., Hussain, M. A., Hussain, Z., Kazmi, S. N. & Bukhari, S. N. A. (2017). *Chem. Biol. Drug Des.* **90**, 443–449. 10.1111/cbdd.12964 28186369

[bb23] Radić, Z., Dale, T., Kovarik, Z., Berend, S., Garcia, E., Zhang, L., Amitai, G., Green, C., Radić, B., Duggan, B. M., Ajami, D., Rebek, J. Jr & Taylor, P. (2013). *Biochem. J.* **450**, 231–242. 10.1042/BJ20121612 PMC4772673 23216060

[bb24] Rigaku OD (2017). *CrysAlis* PRO. Rigaku Oxford Diffraction, Yarnton, England.

[bb25] Sheldrick, G. M. (2015*a*). *Acta Cryst.* A**71**, 3–8.

[bb26] Sheldrick, G. M. (2015*b*). *Acta Cryst.* C**71**, 3–8.

[bb27] Smith, A. G., Tasker, P. & White, D. J. (2003). *Coord. Chem. Rev.* **241**, 61–85.

[bb28] Sørensen, M., Neilson, E. H. J. & Møller, B. L. (2018). *Mol. Plant.* **11**, 95–117. 10.1016/j.molp.2017.12.014 29275165

[bb29] Spackman, M. A. & Jayatilaka, D. (2009). *CrystEngComm*, **11**, 19–32.

[bb30] Spackman, M. A. & McKinnon, J. J. (2002). *CrystEngComm*, **4**, 378–392.

[bb31] Spek, A. L. (2009). *Acta Cryst.* D**65**, 148–155. 10.1107/S090744490804362X PMC2631630 19171970

[bb32] Voicu, V. A., Thiermann, H., Rădulescu, F. Ş., Mircioiu, C. & Miron, D. S. (2010). *Basic Clin. Pharmacol. Toxicol.* **106**, 73–85. 10.1111/j.1742-7843.2009.00486.x 19961476

[bb33] White, F., Forgan, R. & Tasker, P. (2015*b*). Private communication (refcode 1410307). CCDC, Cambridge, England.

[bb34] White, F., Gordon, R. & Tasker, P. (2015*a*). Private communication (refcode 1410312). CCDC, Cambridge, England.

[bb35] Wolff, S. K., Grimwood, D. I., McKinnon, J. J., Turner, M. J., Jayatilaka, D. & Spackman, M. A. (2012). *Crystal Explorer*. The University of Western Australia.

[bb36] Wood, P. A., Forgan, R. S., Henderson, D., Lennie, A. R., Parsons, S., Pidcock, E., Tasker, P. A. & Warren, J. E. (2008*a*). *CrystEngComm*, **10**, 259–251.

[bb37] Wood, P. A., Forgan, R. S., Henderson, D., Parsons, S., Pidcock, E., Tasker, P. A. & Warren, J. E. (2006). *Acta Cryst.* B**62**, 1099–1111. 10.1107/S0108768106031752 17108665

[bb38] Wood, P. A., Forgan, R. S., Lennie, A. R., Parsons, S., Pidcock, E., Tasker, P. A. & Warren, J. E. (2008*b*). *CrystEngComm*, **10**, 239–251.

[bb39] Yadav, P., Lal, K., Rani, P., Mor, S., Kumar, A. & Kumar, A. (2017). *Med. Chem. Res.* **26**, 1469–1480.

[bb40] Zhao, S. Y., Li, K., Jin, Y. & Lin, J. (2018). *Eur. J. Med. Chem.* **144**, 41–51. 10.1016/j.ejmech.2017.12.016 29247859

